# The Extract of *Sonneratia apetala* Leaves and Branches Ameliorates Hyperuricemia in Mice by Regulating Renal Uric Acid Transporters and Suppressing the Activation of the JAK/STAT Signaling Pathway

**DOI:** 10.3389/fphar.2021.698219

**Published:** 2021-08-16

**Authors:** Yu-Lin Wu, Jin-Fen Chen, Lin-Yun Jiang, Xiao-Li Wu, Yu-Hong Liu, Chang-Jun Gao, Yan Wu, Xiao-Qing Yi, Zi-Ren Su, Jian Cai, Jian-Nan Chen

**Affiliations:** ^1^ Guangdong Provincial Key Laboratory of New Drug Development and Research of Chinese Medicine, School of Pharmaceutical Sciences, Guangzhou University of Chinese Medicine, Guangzhou, China; ^2^ The First Affiliated Hospital of Chinese Medicine, Guangzhou University of Chinese Medicine, Guangzhou, China; ^3^ School of Biomedical and Pharmaceutical Sciences, Guangdong University of Technology, Guangzhou, China; ^4^ Guangdong Academy of Forestry, Guangzhou, China; ^5^ Guangdong Provincial Key Laboratory of Silviculture, Protection and Utilization, Guangzhou, China

**Keywords:** *Sonneratia apetala* leaves and branches, hyperuricemia, renal uric acid transporters, oxidative stress, JAK/STAT pathway

## Abstract

*Sonneratia apetala* Buch-Ham., an exotic mangrove species with antidiabetic, antibacterial, and antioxidant capacities, mainly distributes in the southeast coastal areas in China. The present work investigated the protective effects of *Sonneratia apetala* leaves and branches extraction (SAL) on hyperuricemia (HUA) in mice. Potassium oxonate (PO) and hypoxanthine (HX) were used to establish the HUA model by challenge for consecutive 7 days. Results revealed that SAL inhibited the increases in kidney weight and index compared to the vehicle group. Meanwhile, SAL significantly decreased the levels of uric acid (UA), creatinine (CRE), and blood urea nitrogen (BUN) in serum. Additionally, SAL inhibited the activity of xanthine oxidase (XOD) in the liver. SAL ameliorated PO- and HX-induced histopathological changes. Moreover, it regulated oxidative stress markers including malondialdehyde (MDA), catalase (CAT), superoxide dismutase (SOD) activity, and glutathione (GSH) content. Also, SAL inhibited the increases in renal levels of interleukin-6 (IL-6), interleukin-18 (IL-18), interleukin-1β (IL-1β), tumor necrosis factor (TNF-α), monocyte chemotactic protein 1 (MCP-1), and transforming growth factor-β (TGF-β). SAL remarkably reduced suppressor of cytokine signaling 3 (SOCS3), Janus kinase 2 (JAK2), and subsequent phosphorylation of signal transducer and activator of transcription 3 (STAT3) expression. In addition, SAL inhibited the activation of nuclear factor kappa-B (NF-κB) in the kidney. Furthermore, SAL protected against HUA by regulating renal UA transporters of organic anion transporter (OAT1), urate reabsorption transporter 1 (URAT1), and glucose transporter 9 (GLUT9). These findings suggested that SAL ameliorated HUA by inhibiting the production of uric acid and enhancing renal urate excretion, which are related to oxidative stress and inflammation, and the possible molecular mechanisms include its ability to inhibit the JAK/STAT signaling pathway. Thus, SAL might be developed into a promising agent for HUA treatments.

## Introduction

Hyperuricemia (HUA) is a metabolic disease caused by abnormal purine metabolism or insufficient uric acid (UA) excretion, characterized by elevated UA in the blood (Borghi, 2017; Dehlin et al., 2020). Previous studies report that HUA is highly related to hypertension, hyperlipidemia, fatty liver, diabetes, and other diseases (Wang et al., 2014; Jaruvongvanich et al., 2017; Jayachandran and Qu, 2020; Kielstein et al., 2020). With improvement of people’s living standard and change of dietary pattern, incidence of HUA is increasing in recent years (Liu et al., 2014; Singh et al., 2019). Hepatic overproduction and renal underexcretion of uric acid are two of the main causes of HUA (Shekelle et al., 2017). UA production is catalyzed by xanthine oxidase (XOD) in the liver, whereas excretion of UA occurs in the kidney. XOD is the key enzyme involved in the transformation of xanthine and hypoxanthine (HX) into UA, which is distributed in the liver (Maiuolo et al., 2016). On the other hand, during the process of UA excretion, urate transporters play a vital role in reabsorption and secretion of UA (Pan et al., 2020). Moreover, accumulation of UA in the kidney can lead to UA crystals and cause inflammation and oxidative stress, thereby leading to kidney injury (Chang et al., 2014). In conclusion, reducing UA production, promoting UA excretion, or (and) ameliorating the inflammation and oxidative stress may be the potential therapeutic methods of HUA.

In clinical practice, treatment of HUA is mainly achieved by facilitating UA excretion or inhibiting XOD activity. The first-line XOD inhibitors are allopurinol (AP) and febuxostat (FBX) (Shekelle et al., 2017). However, less than 40% of patients can reduce their serum UA levels to normal levels by taking AP, and long-term use of AP can lead to liver and kidney injury (Yang et al., 2015). Research also reported that patients treated with FBX faced higher risk of cardiovascular death (Zhang et al., 2018). In addition, as a transporter inhibitor, benzbromarone (BZM) is also a first-line drug against HUA (Dong et al., 2019). Nevertheless, long-term use of BZM was reported to cause serious hepatotoxicity, which limited its clinical application (Shin et al., 2011). Therefore, it is necessary to explore natural and nontoxic therapeutic strategies for HUA. Mounting pieces of evidence have suggested that natural medicine exhibited significant effects in lowering UA (Chen et al., 2015; Liang et al., 2019; Martins de Sá Müller et al., 2019). Therefore, there is great potential for natural medicine to be developed as an alternative drug against HUA in the future.

*Sonneratia apetala* Buch-Ham (*S. apetala*), an exotic member of the mangrove, widely distributes in the coastal regions of Bangladesh, India, China etc. (Patra et al., 2015; Hossain S. J. et al., 2016; Hossain S. J. et al., 2016). Most of mangrove plants have strong pharmacological activities due to the special growth environment. *S. apetala* is extensively used by the indigenous people for diuretic, antidiabetic, catharsis, and sprains (Hossain S. J. et al., 2016; Sachithanandam et al., 2019). Polysaccharides, polyphenols, and flavonoids were rich in *S. apetala* (Bandaranayake, 2002; Rahmatullah et al., 2010; Teja and Ravishankar, 2013; Patra et al., 2015). Previous study suggested that *S. apetala* possessed potential abilities of free radical scavenging and reducing power (Mukul et al., 2016).

In our preliminary experiment, components of *S. apetala* leaves and branches (SAL) were identified by HPLC-MS. The result showed that the main components of SAL were gallic acid, isorhamnetin, and vitexin. In the previous study, gallic acid was demonstrated to have a significant inhibitory effect on XOD *in vitro* (Lespade and Bercion, 2010). Moreover, vitexin could inhibit the production of interleukin-1β (IL-1β), tumor necrosis factor (TNF-α), and nitric oxide (NO) in macrophage RAW 267.4 cells challenged by lipopolysaccharides (LPS) (Rosa et al., 2016). Furthermore, isorhamnetin has been reported to show anti-hyperuricemia effects (Adachi et al., 2019). Therefore, SAL might exert a favorable anti-hyperuricemia effect through the hypouricemic, antioxidant, and anti-inflammatory properties of these components. To our knowledge, there are no reports on the chemical components of SAL and protective effect of SAL on HUA. Thus, it was the first study to investigate the protective effect of SAL against PO- and HX-induced HUA. For the first time, SAL was demonstrated to possess anti-hyperuricemia effect. Through further analysis, the signaling pathway against HUA was clarified. Additionally, the experiment is beneficial for utilization and protection of mangrove plants. The results outlined might add new dimension to the anti-hyperuricemia effect and mechanism of *S. apetala*, which may arouse interest in its further investigation.

Therefore, the aim of the current study was to investigate the protective effects of SAL on HUA in mice. To further clarify the mechanisms, effects of SAL on renal urate transporters, the Janus kinase (JAK)/signal transducer and activator of transcription (STAT) pathway was investigated through Western blot and quantitative real-time polymerase chain reaction (qPCR) analysis.

## Materials and Methods

### Materials and Chemicals

*S. apetala* leaves and branches were obtained from Nansha Coast Wetland in Guangzhou, China (2017GDZJ011). Potassium oxonate (97%, 2207-75-2, PO) and HX (99%, 68-94-0) were purchased from Sigma-Aldrich (United States). FBX (98%, 144060-53-7) was from Shanghai Yuanye Bio-Technology Co. LTD. (Shanghai, China). The biochemical assay kits of UA (C012-2), creatinine (CRE, C011-2-1), blood urea nitrogen (BUN, C013-2-1), glutathione (GSH, A006-2), catalase (CAT, A007-1-1), superoxide dismutase (SOD, A001-3), malondialdehyde (MDA, A003-1), and XOD (A002-1-1) were the products of Nanjing Jiancheng Bioengineering Institute (Nanjing, China). The antibodies against JAK2 (ab108596), p-JAK2 (ab195055), STAT3 (ab68153), p-STAT3 (ab131103), and β-actin (ab8227) were obtained from Abcam Biosciences (Inc., United States). Glucose transporter 9 (GLUT9, 26486-1-AP) was purchased from Protein technology Co. LTD. (Inc., United States). Organic anion transporter (OAT1, DF6633), urate reabsorption transporter 1 (URAT1, DF12340), nuclear factor kappa-B P65 (NF-κB P65, AF5006), anti-histone H3 antibodies (AF0863), and secondary antibodies (110191) were obtained from Affinity Biosciences (Inc., United States). Other chemicals and reagents were obtained from local suppliers.

### Extraction of SAL

Fresh *S. apetala* leaves and branches were rinsed and extracted in boiling distilled water twice for 5 h. After filtration, the extracting solution was concentrated by reducing pressure and then freeze-dried to obtain SAL (Liu et al., 2019).

### Chemical Compositions Analysis

To determine the components of SAL, the ultra-performance liquid chromatography (UPLC)/qExactive-mass spectrum (MS) method was performed (Hossain S. J. et al., 2016). In brief, SAL was dissolved in 80% methanol, centrifuged (20,000 × *g*, 4°C, 10 min), and the filtered with millipore filters (0.22 μm). The filtrate was injected for UPLC analysis through an RP-C18 column (2.1 mm × 150 mm, 1.8 μm; Welch) at 35°C. The mobile phases were 0.1% (*v/v*) formic acid dissolved in purified water (A) and 0.1% (*v/v*) formic acid dissolved in acetonitrile (B). The elution program was set with a gradient procedure as follows: 0–1 min, 2% B; 1–10 min, 2–50% B; 10–20 min, 50–95% B; 20–25 min, 95% B; 25–26 min, 95–2% B; and 26–30 min, 2% B. The injection volume of SAL was 5 μL and eluted at 0.3 ml/min. The MS full scan range was 150–2,000 m/z. The collision gas and desolvation gas were high-purity N_2_ and Ar, respectively. Finally, the results were compared across the databases (mzCloud, mzVault, and ChemSpider).

### Animal Experiments

Eight-week-old male Kunming mice were supplied by Guangdong Medical Laboratory Animal Center (Guangzhou, China, No.44007200077071). All the mice were acclimated for one week with a 12-h light/dark cycle, a controlled temperature (22 ± 2°C), and a relative humidity of 55 ± 5% before experiment. The mice were allowed to obtain food and water freely. The experiment was conducted under the supervision of the Laboratory Animal Ethics Committee of Guangzhou University of Chinese Medicine [SYXK (Yue) 2018-0085] and in accordance with Regulations for the Administration of Affairs Concerning Experimental Animals (Ethics NO.20200602006).

In brief, mice were randomly divided into seven groups (*n* = 10): intact, vehicle, BZM (10 mg/kg), FBX (10 mg/kg), and SAL groups (50, 100, and 200 mg/kg) (Liu et al., 2019). Except those in the intact group, all the mice were intragastrically given HX (300 mg/kg) and intraperitoneally injected with PO (300 mg/kg) (Lu et al., 2019). One h after administration with PO and HX, mice in SAL-treated groups, the BZM group, and the FBX group were given a corresponding dose of SAL, BZM, or FBX, while those in the intact and vehicle groups were fed with equal volumes of 0.5% of carboxymethylcellulose sodium (CMC-Na) solution by gastric gavage. All the operations were conducted once daily for consecutive 1 week. After the last treatment, all the mice were fasted for 4 h. Subsequently, the mice were anesthetized using 3% of pentobarbital sodium (Jiewei et al., 2018). Blood samples were obtained from the orbit and centrifuged for 10 min (1,000 rpm, 4°C) after clotting at room temperature for 120 min. Serum was obtained and stored at −80°C for the further analysis. Afterward, all the animals were sacrificed, and the kidney tissues were weighed and collected for following biochemistry analysis. The kidney index was calculated according to the following formula: the kidney index of the mice = (kidney weight of the mice/body weight of the mice) × 100%.

### Kidney Histopathology

Fresh kidney tissues were rinsed thoroughly, fixed, and then embedded in paraffin. Subsequently, 5 -mμ tissues were cut and finally dyed with hematoxylin and eosin (H and E) or periodic acid-Schiff (PAS) routinely. Finally, kidney histopathological changes were examined using a microscope at × 200 magnification.

### Reactive Oxygen Species Assays

To detect the level of ROS in the kidney, dihydroethidium (DHE) staining was adopted according to the methods, as previously described (Semprun-Prieto et al., 2011). Fresh kidney tissues were prepared into 5-μm cryosections. The slices were then incubated with the DHE (100 μmol/L) in the dark for 30 min at 37°C. The results were quantified as fluorescence intensity.

### Serum Biochemical Assays

The serum of the mice was obtained by centrifugation (3,500 rpm, 10 min, and 4°C) after being kept at 25°C for 2 h. Then the activities of serum UA, CRE, and BUN were measured by respective commercials kits.

### XOD Activity Assay

The liver tissues were homogenized and centrifuged at 3,000 rpm for 10 min (4°C). Subsequently, the supernatant was used for the measurement of XOD activity by a commercial kit.

### Kidney Biochemical Assays

The supernatant of renal homogenate was obtained by the method of 2.8. Subsequently, under instruction of manufacture’s protocols, the renal GSH, MDA levels, and SOD, CAT activities were measured by using commercial kits.

### Enzyme-Linked Immunosorbent Assays

Following the manufacturer’s instructions, the levels of interleukin-6 (IL-6), interleukin-18 (IL-18), IL-1β, and TNF-α in the kidney were detected by using ELISA kits obtained from MLBIO Biotechnology Co., Ltd. (Shanghai, China).

### Quantitative Real-Time Polymerase Chain Reaction

To isolate the total ribonucleic acid (RNA) from kidneys of the mice, Trizol reagent was used according to manufacturer’s instruction. The RNA purity was identified to be between 1.8 and 2.0. The obtained RNA was then reverse-transcribed into cDNA. The mRNA expressions of IL-1β, IL-6, IL-18, TNF-α, monocyte chemotactic protein 1 (MCP-1), transforming growth factor-β (TGF-β), suppressor of cytokine signaling 3 (SOCS3), and glyceraldehyde-3-phosphate dehydrogenase (GAPDH) were determined by using HiScript^®^ II Q RT SuperMix (+ gDNA wiper) and ChamQ™ SYBR^®^ qPCR Master Mix Kit (Livak and Schmittgen, 2001). The sequences of primers, shown in Table 1, were designed by online primer design software (Sangon Biotech Co., LTD., Shanghai). The relative quantifications of genes expression were calculated using the 2^−ΔΔCq^ method with GAPDH served as a normalization control.

**TABLE 1:** Primer sequences.

Targeted gene	Direction and sequence (5′ to 3′)
IL-1β	Forward: TCG​CAG​CAG​CAC​ATC​AAC​AAG​AG
	Reverse: TGC​TCA​TGT​CCT​CAT​CCT​GGA​AGG
IL-6	Forward: CTT​CTT​GGG​ACT​GAT​GCT​GGT​GAC
	Reverse: AGG​TCT​GTT​GGG​AGT​GGT​ATC​CTC
IL-18	Forward: CAA​AGT​GCC​AGT​GAA​CCC​CAG​AC
	Reverse: ACA​GAG​AGG​GTC​ACA​GCC​AGT​C
TNF-α	Forward: GCC​TCT​TCT​CAT​TCC​TGC​TTG​TGG
	Reverse: GTG​GTT​TGT​GAG​TGT​GAG​GGT​CTG
MCP-1	Forward: CCA​CTC​ACC​TGC​TGC​TAC​TCA​TTC
	Reverse: CTT​CTT​TGG​GAC​ACC​TGC​TGC​TG
TGF-β	Forward: CAG​GCT​CTG​GAG​AAC​AGC​ACA​TC
	Reverse: TGG​GAA​TCT​GGG​CAC​TTG​TTG​AAG
SOCS3	Forward: GAC​CAA​GAA​CCT​ACG​CAT​CCA​GTG
	Reverse: GCA​CCA​GCT​TGA​GTA​CAC​AGT​CG
GAPDH	Forward: GGT​TGT​CTC​CTG​CGA​CTT​CA
	Reverse: TGG​TCC​AGG​GTT​TCT​TAC​TCC

### Western Blot Analysis

The kidney tissues were extracted with protein lysis buffer containing phenylmethanesulfonyl fluoride (PMSF) and protease inhibitor cocktail to obtain the total protein. Besides, extraction of cytoplasmic and nucleus protein was performed using the extraction kit (Thermo). The protein concentration was measured by the Bicinchoninic Acid (BCA) Protein Assay Kit. Sodium dodecyl sulfate–polyacrylamide gel electrophoresis (SDS-PAGE) was used to separate the protein samples, which were then transferred onto a polyvinylidene fluoride (PVDF) membrane. After blocking with 5% skimmed milk, the membranes were incubated with primary antibody and a horseradish peroxidase (HRP) goat anti-rabbit antibody (Abo-Youssef et al., 2020). The bands were detected by electrochemiluminescence (ECL) reagent. The density of each band was analyzed using ImageJ.

### Statistical Analysis

All the data were expressed as mean ± standard deviation (SD), and statistical analysis was performed by SPSS software 23.0. The data were analyzed by one-way analysis of variance (ANOVA) followed by Dunnett’s test. The figures were processed using GraphPad Prism 8.0.1. The value of *p* <0.05 was regarded as statistical significance.

## Results

### Chemical Composition of SAL

**FIGURE 1:**
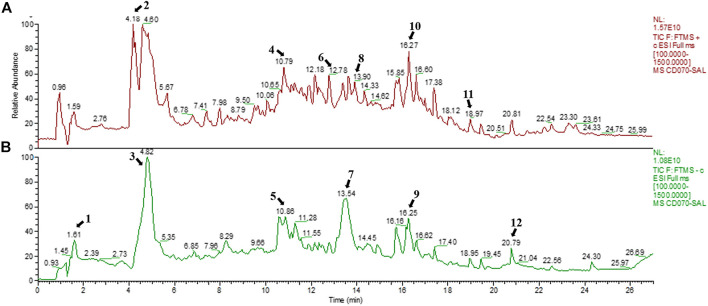
Chemical composition of SAL. **(A)** Positive mode; **(B)** negative mode.

SAL was obtained from *S. apetala* leaves by boiling water extraction, achieving a yield of 4.23%. The positive and negative ion chromatograms of SAL are shown in Figure 1, and the characterization of the compounds is presented in Table 2.

**TABLE 2:** Identification of the chemical constituents in SAL.

Number	Retention time (min)	Ion mode	Extraction mass (Da)	Found mass (Da)	Error (ppm)	Formula	Identification	Peak area (%)
1	1.61	−	134.0200	134.0201	0.6715	C_4_H_6_O_5_	L-(-)-Malic acid	0.7299
2	4.18	+	153.1159	153.1152	−4.9636	C_9_H_17_NO_2_	(4E)-3-Hydroxy-2,4-dimethyl-4-heptenamide	8.8478
3	4.82	−	170.0205	170.0203	−1.5880	C_7_H_6_O_5_	Gallic acid	9.8934
4	10.79	+	178.0994	178.0992	−0.7861	C_11_H_14_O_2_	4-Isobutylbenzoic acid	0.2395
5	10.86	−	335.1158	335.1153	−1.3428	C_20_H_17_NO_4_	Berberine	0.1830
6	12.75	+	150.1045	150.1044	−0.5330	C_10_H_14_O	Carvone	0.1451
7	13.54	−	316.0583	316.0582	−0.2848	C_16_H_12_O_7_	Isorhamnetin	6.5621
8	13.89	+	432.1057	432.1052	−1.1571	C_21_H_20_O_10_	Vitexin	1.8300
9	16.25	−	132.0575	132.0577	1.5145	C_9_H_8_O	trans-Cinnamaldehyde	0.1165
10	16.29	+	314.2457	314.2454	−1.1456	C_18_H_34_O_4_	(+/−)12(13)-DiHOME	2.8721
11	18.99	+	350.2063	350.2064	0.2570	C_20_H_30_O_5_	Andrographolide	0.2457
12	21.40	−	337.3345	337.3337	−2.2233	C_22_H_43_NO	Erucamide	0.3520

### Effects of SAL on Renal Histopathology

**FIGURE 2:**
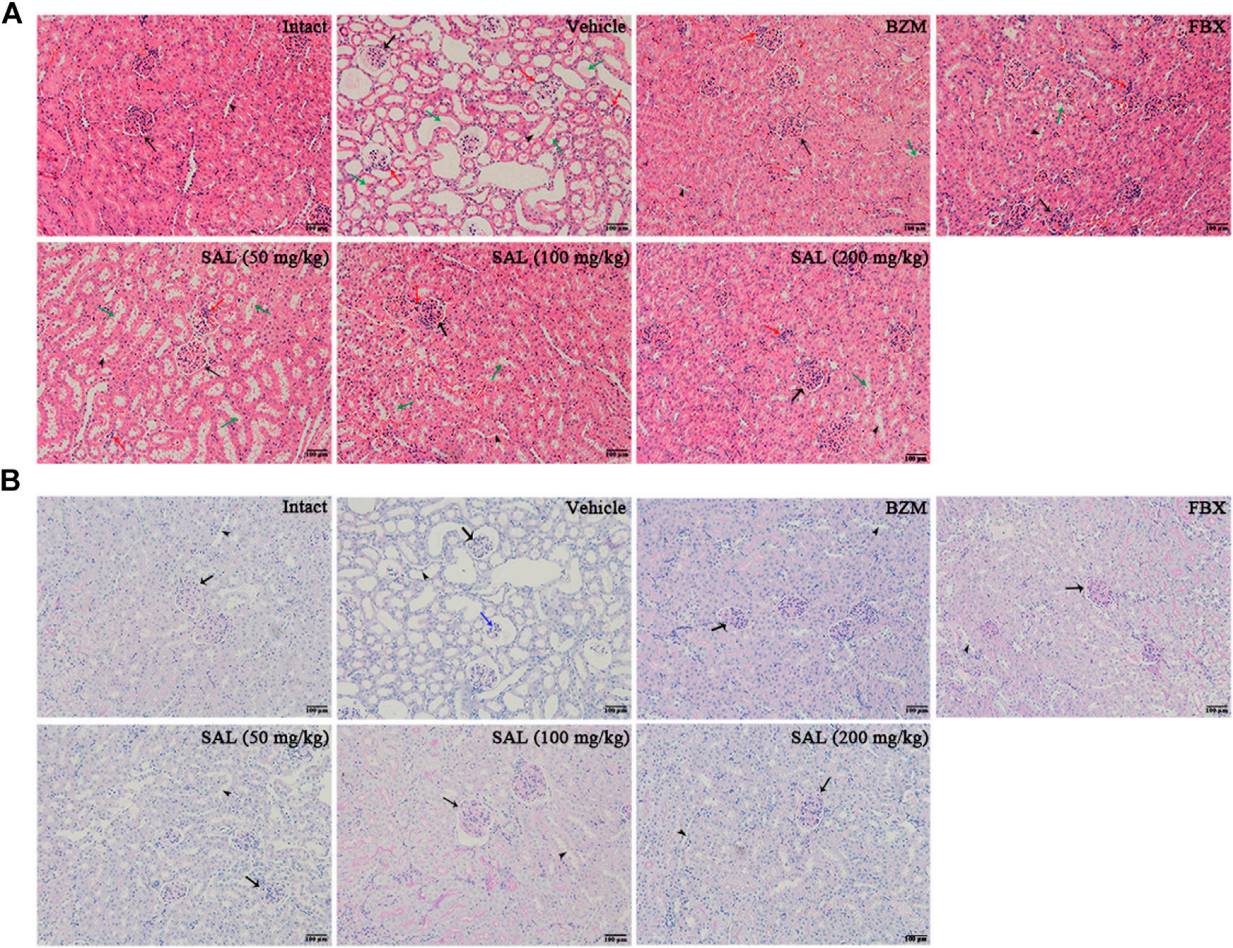
Effects of SAL on renal histopathology. **(A)** H and E; **(B)** PAS. Magnification: × 200. Scale bars: 100 μm. Black arrows: glomerulus; sessile arrows: renal tubules; green arrows: epithelial cells exfoliation; red arrows: inflammatory infiltration; blue arrows: incrassated basement membrane.

To investigate the effect of SAL on renal morphological changes in HUA mice, H and E and PAS staining were performed. As shown in results of H and E staining (Figure 2A), the morphology of the kidney cells in the intact group was in a healthy condition. The glomeruli, renal tubules, renal cortex, and medulla were clearly structured. In contrast, the HUA mice showed inflammatory cellular infiltration, along with obvious edema, necrosis, and balloon-like changes in the glomeruli, surrounding renal tubules and renal tubular epithelial cells. The appearance of kidneys in the vehicle group confirmed that the HUA model was established successfully. BZM and FBX treatment reduced the degree of edema and diminished the inflammatory cellular infiltration in the renal interstitium significantly. Moreover, all the renal pathological changes were also ameliorated by the SAL in a dose-dependent manner.

As presented in results of PAS staining (Figure 2B), the glomeruli basement membrane of HUA mice was significantly thickened compared with the intact group. The BZM and FBX treatment partially attenuated the thickened basement membrane. Moreover, the SAL treatment mitigated the thickening of glomeruli basement membrane dose-dependently.

### Effect of SAL on the UA, CRE, and BUN Levels in Serum

**FIGURE 3:**
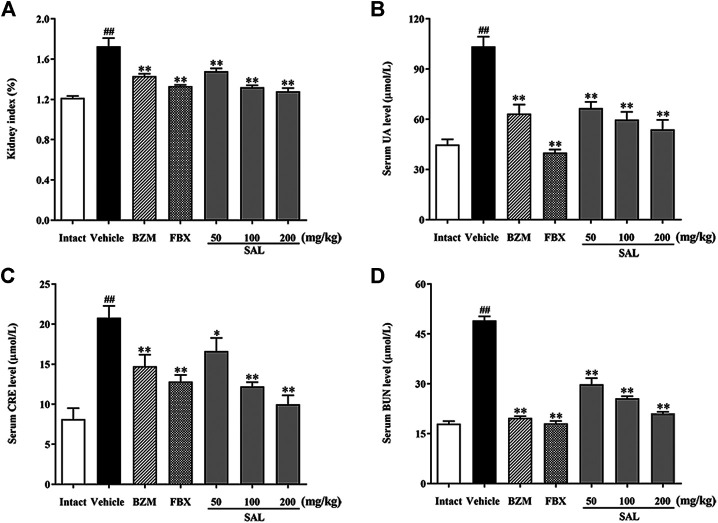
Effect of SAL on the levels of UA, CRE, and BUN in serum. **(A)** Kidney index; level of **(B)** UA; **(C)** CRE; **(D)** BUN in serum. All data are expressed as mean ± SD (*n* = 8). ^##^ *p* < 0.01 vs. the intact group, *p < 0.05, **p < 0.01 vs. the vehicle group.

To validate the effects anti-hyperuricemia and nephroprotective effects of SAL in HUA mice, UA, CRE, and BUN levels in serum were determined. As shown in Figures 3B–D, the levels of serum UA, CRE, and BUN in the vehicle group were significantly (*p* < 0.01, *p* < 0.01, and *p* < 0.01, respectively) increased compared to those in the intact group, which suggested that the HUA model was established successfully. The renal index in the vehicle group was also increased markedly (*p* < 0.01). BZM significantly decreased the levels of serum UA, CRE, and BUN (*p* < 0.01, *p* < 0.01, and *p* < 0.01, respectively). FBX also decreased the levels of UA, CRE, and BUN in serum (*p* < 0.01, *p* < 0.01, and *p* < 0.01, respectively). When compared with the vehicle group, SAL at 50, 100, and 200 mg/kg also reduced the levels of UA, CRE, and BUN dose-dependently.

### Effect of SAL on the Activity of XOD in Liver

**FIGURE 4:**
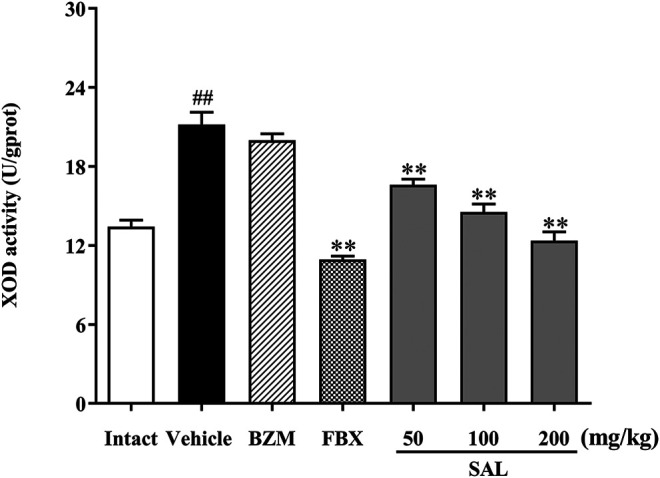
Effect of SAL on the activity of XOD in liver. All data are expressed as mean ± SD (*n* = 8). ^##^ *p* < 0.01 vs. the intact group, **p < 0.01 vs. the vehicle group.

To illuminate whether SAL could affect uric production in mice, XOD activity in liver was measured. As shown in Figure 4, hepatic XOD activity of the vehicle group was markedly elevated in mice (*p* < 0.01) compared to the intact group. On the contrary, FBX remarkedly inhibited the XOD activity in HUA mice (*p* < 0.01). However, BZM showed no inhibitory effect (*p* > 0.05) on XOD activity. Surprisingly, SAL attenuated the elevated XOD activity in the liver at 50, 100, and 200 mg/kg (*p* < 0.01 for all). Therefore, SAL might ameliorate HUA by suppressing UA production.

### Effect of SAL on Oxidative Stress

**FIGURE 5:**
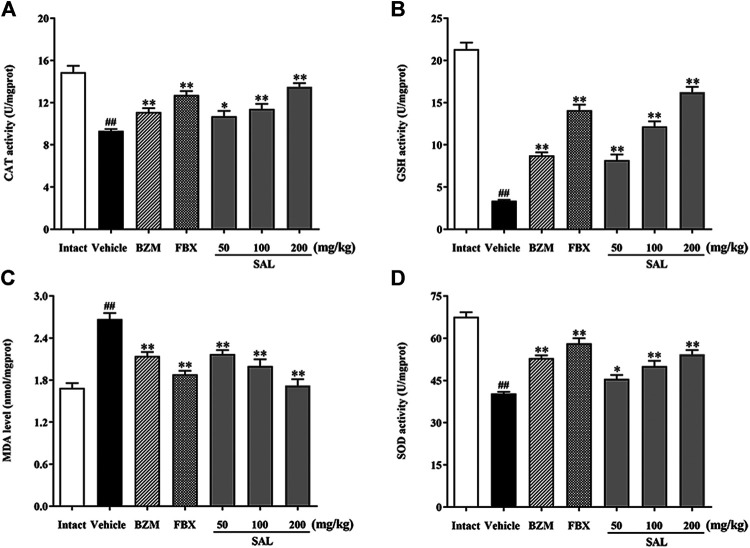
Activity of antioxidant enzymes. **(A)** CAT; **(B)** GSH; **(C)** MDA; **(D)** SOD. All data are expressed as mean ± SD (*n* = 8). ^##^ *p* < 0.01 vs. the intact group, *p < 0.05, **p < 0.01 vs. the vehicle group.

**FIGURE 6:**
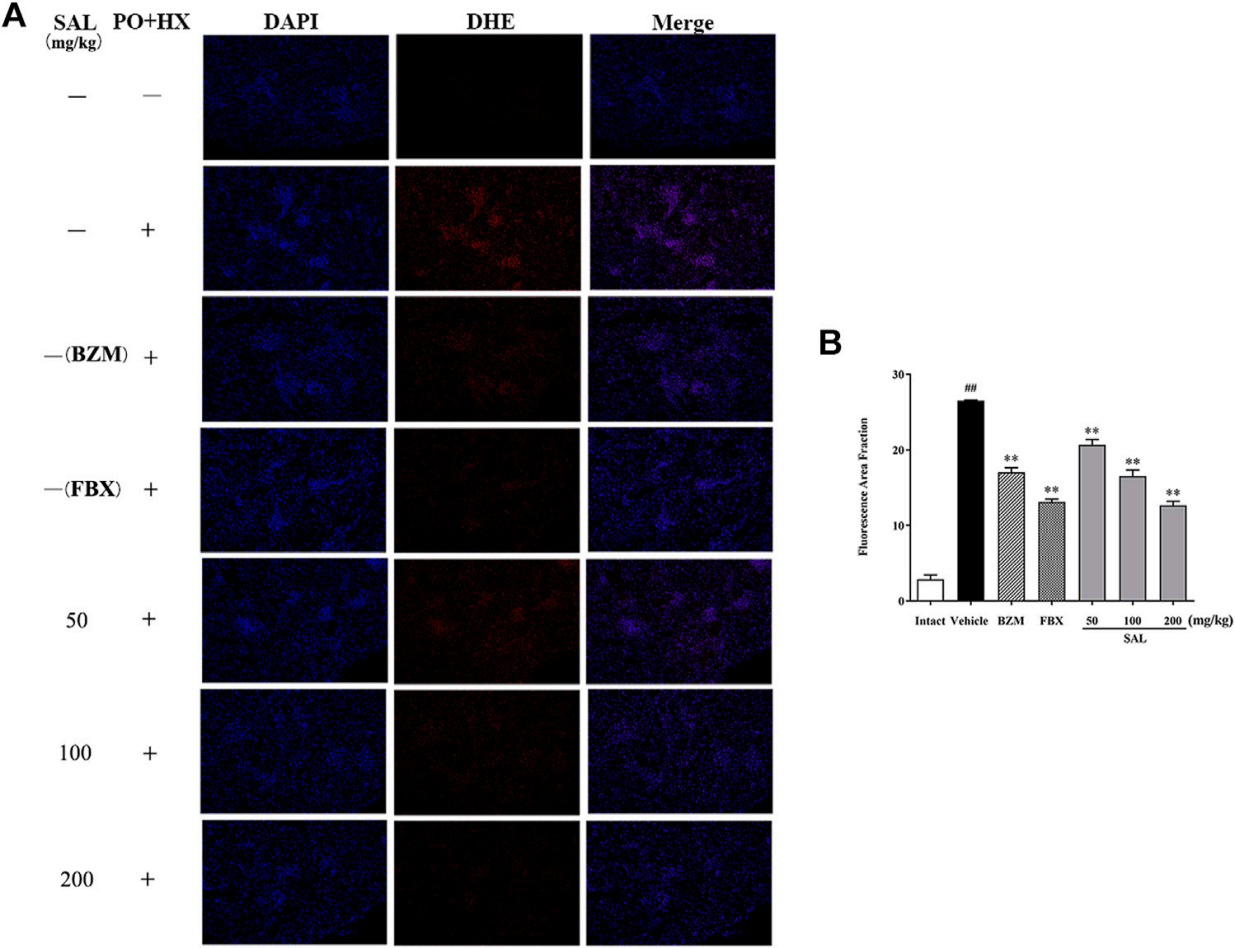
Effects of SAL on markers of oxidative stress. **(A)** DHE staining of the renal tissues; **(B)** fluorescence area intensity. All data are expressed as mean ± SD (*n* = 3). ^##^ *p* < 0.01 vs. the intact group, **p < 0.01 vs. the vehicle group.

To evaluate ameliorative effect of SAL on renal oxidative stress in the HUA mice, activities of antioxidant enzymes were analyzed. As shown in Figure 5, compared with those of the intact group, the renal GSH level and SOD and CAT activity decreased significantly, while the MDA level increased significantly in the vehicle group. However, the BZM and FBX treatment could restore the renal SOD, CAT activity, MDA, and the GSH level in the HUA mice. Moreover, administration with SAL significantly increased the activities of SOD, GSH, and CAT (*p* < 0.01 for all) and reduced the level of MDA, especially at 100 and 200 mg/kg (*p* < 0.01 for both), suggesting that SAL could suppress oxidative stress in the HUA mice. In addition, the ROS level markedly elevated in the vehicle group compared with the intact group and decreased in both BZM- and FBX-treated groups (Figure 6). Meanwhile, SAL at 50, 100, and 200 mg/kg decreased the ROS intensity significantly (*p* < 0.01 for all), which showed obvious inhibitory effects on ROS production. Therefore, these results indicated that SAL might suppress renal oxidative stress in HUA mice.

### Effect of SAL on Renal Inflammatory Cytokines

**FIGURE 7:**
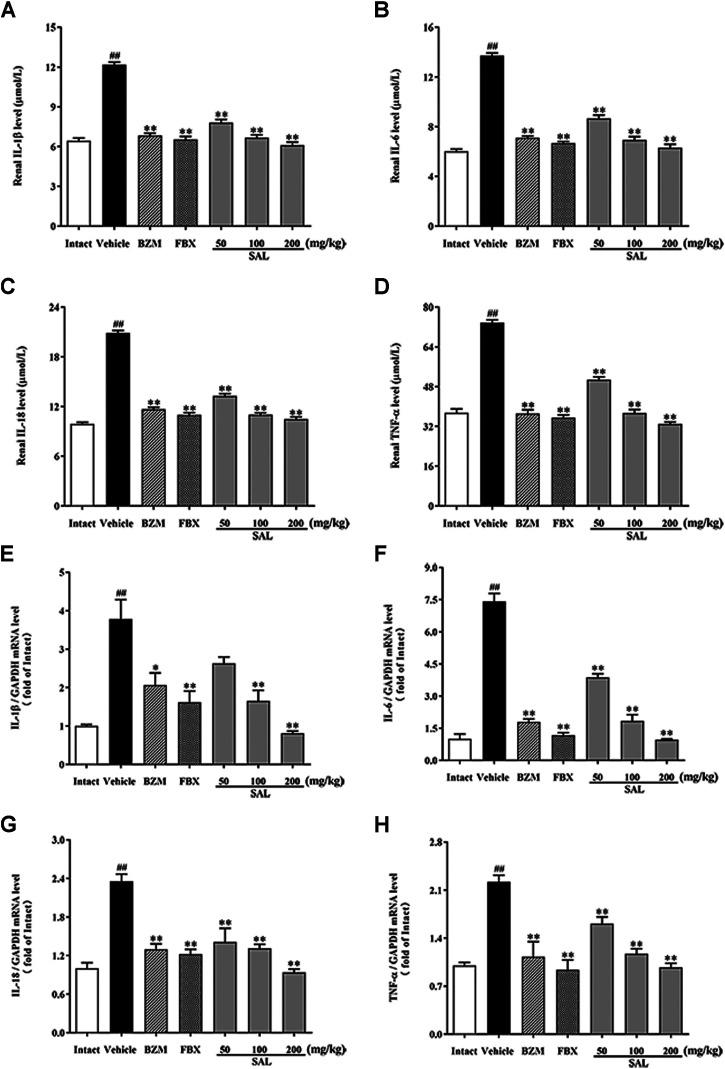
Effect of SAL on renal inflammatory cytokines. Renal **(A)** IL-1β; **(B)** IL-6; **(C)** IL-18; **(D)** TNF-α; mRNA expression of **(E)** IL-1β; **(F)** IL-6; **(G)** IL-18; **(H)** TNF-α. All data are expressed as mean ± SD (*n* = 8). ^##^ *p* < 0.01 vs. the intact group, **p < 0.01 vs. the vehicle group.

To further investigate whether SAL could also suppress renal inflammation *in vivo*, levels of inflammatory cytokines involving IL-1β, IL-6, IL-18, and TNF-α were determined by ELISA and RT-PCR. Remarkable elevated levels of the renal inflammatory cytokines were observed in the vehicle group (*p* < 0.01 for all). However, treatment with SAL significantly decreased the levels of these inflammatory cytokines in the kidneys of the HUA mice. Of note, SAL at 200 mg/kg exhibited the same effect on reducing the levels of the inflammatory cytokines compared to the BZM and FBX treated groups (Figure 7).

### Effects of SAL on the NF-κB/JAK/STAT Signaling Pathway

**FIGURE 8:**
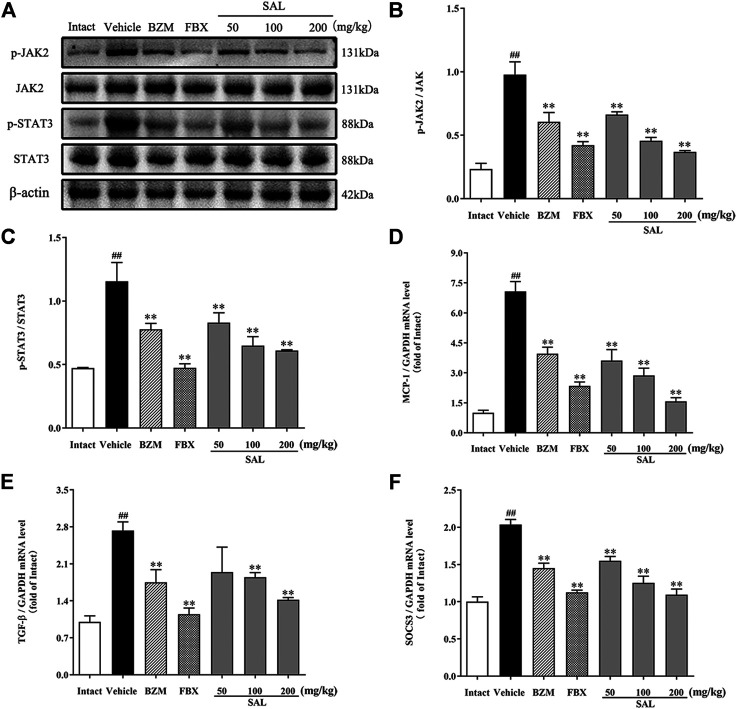
Effects of SAL on the JAK/STAT signal pathway. **(A)** The expression levels of the JAK2, p-JAK2, STAT3, and p-STAT3; quantitative results of Western blot analyses for **(B)** p-JAK2/JAK2; **(C)** p-STAT3/STAT3; relative mRNA expression of **(D)** MCP-1; **(E)** TGF-β; and **(F)** SOCS3. β-actin was used as a loading control. All data are expressed as mean ± SD (*n* = 3). ^##^ *p* < 0.01 vs. the intact group, **p < 0.01 vs. the vehicle group.

**FIGURE 9:**
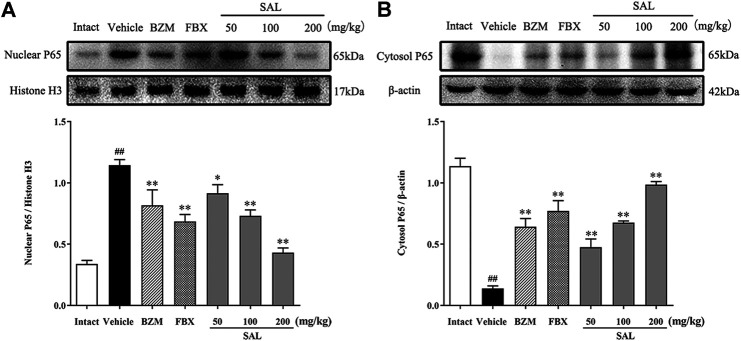
Effects of SAL on the NF-κB signaling pathway. **(A)** Cytosol NF-κB P65; **(B)** Nuclear NF-κB P65.

The JAK/STAT signal pathway is closely related to inflammation that regulates the expression of inflammatory cytokines. As shown in Figure 8, the protein levels of p-JAK2 and p-STAT3 (*p* < 0.01 for both) were significantly increased in HUA mice compared to those in mice from the intact group. Compared to the vehicle group, SAL downregulated renal protein expression of p-JAK2 and p-STAT3 in a dose-dependent manner. Besides, the gene expression of MCP-1, TGF-β, and SOCS3 were increased significantly in the vehicle group. Contrarily, mRNA levels of all these downstream targets were reduced by treatment with BZM, FBX, and SAL (*p* < 0.01 for all). Moreover, after challenge with PO and HX, the protein level of nuclear NF-κB p65 was increased, whereas that of cytosol NF-κB p65 was decreased in the HUA mice (*p* < 0.01 for both). As shown in Figure 9, treatment with BZM, FBX, or SAL attenuated the translocation of NF-κB p65 from the cytoplasm to the nucleus. Furthermore, SAL at 200 mg/kg showed greater effects on reduction of nuclear NF-κB p65 and augmentation of cytosol NF-κB p65 (*p* < 0.01, both) relative to BZM and FBX (Figure 9).

### Effects of SAL on the Expression of Uric Acid Transporters

**FIGURE 10:**
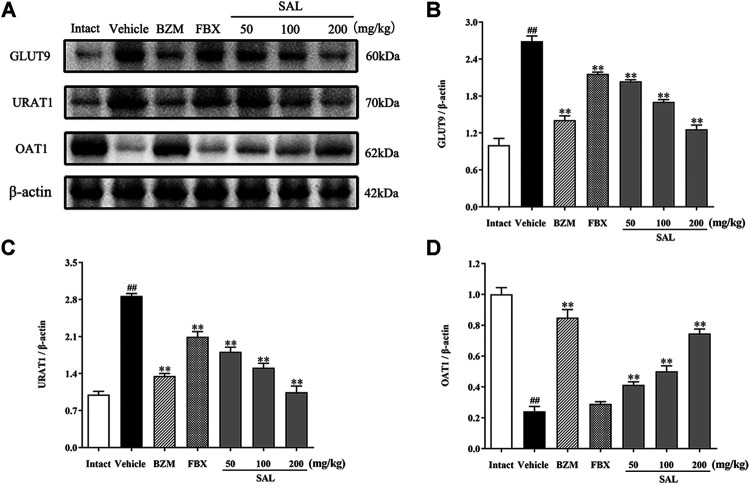
Effects of SAL on the expression of uric acid transporters. **(A)** The expression levels of the URAT1, GLUT9, and OAT1; quantitative results of Western blot analyses for **(B)** GLUT9; **(C)** URAT1; **(D)** OAT1. β-actin was used as a loading control. All data are expressed as mean ± SD (*n* = 3). ^##^ *p* < 0.01 vs. the intact group, **p < 0.01 vs. the vehicle group.

To study whether SAL could directly promote the urate excretion, we measured the protein levels of renal urate transporters by Western blot, which was associated with uric acid reabsorption and excretion (Figure 10). After hyperuricemia induction, the protein levels of URAT1 and GLUT9 were significantly upregulated compared to the intact group (*p* < 0.01 for both), whereas those of OAT1 declined (*p* < 0.01). Treatment with SAL obviously inhibited the elevation of URAT1, GLUT9 expression, and dramatically reversed the decrease in OAT1 expression.

## Discussion

In this study, the phytochemical constituents and the anti-hyperuricemic effect of SAL were demonstrated. PO and HX are widely used to establish the experimental animal models for HUA (Lu et al., 2019). After oral administration of HX and intraperitoneal injection of PO combined for a week, a dramatic increase in the UA level in the vehicle group mice was observed, which suggested the successful establishment of HUA model (Ichida et al., 2012). In addition, the results showed that the HUA mice induced by PO and HX were also accompanied by renal injury. The levels of CRE and BUN were significantly increased, and the kidney index was also significantly increased, suggesting that the function of kidney was damaged. However, SAL remarkably lowered the level of UA *via* HUA mice, confirming the hypouricemic effect of SAL. Additionally, when the kidney is exposed to high UA or damage, increased CRE and BUN levels are regarded as two important biochemical indexes (Wang et al., 2018). Results showed that SAL dose-dependently attenuated the rise of serum CRE and BUN, indicating that SAL could alleviate HUA. Additionally, this study further confirmed the protective effect of SAL on HUA-induced kidney injury through histopathological examination (H and E and PAS staining). The results showed that the renal tubules of the vehicle mice were significantly dilated, and epithelial cells were severely necrotic or even shedding. The glomeruli necrosis was also observed, mostly with atrophy and thickening of the basement membrane. After SAL treatment, the histopathological damage mentioned before were all alleviated. In summary, SAL exerts a favorable anti-HUA effect and has a protective effect on kidney damage caused by HUA.

Generally, the level of UA in the blood is regulated by a balance between UA production and excretion (Kumar et al., 2011; Ichida et al., 2012). HUA occurs when the balance of synthesis and elimination is broken. XOD is the key enzyme for regulating transformation of hypoxanthine and xanthine into UA (Kumar et al., 2011). Clinically, XOD inhibitors, such as allopurinol and FBX, are used as first-line options to treat HUA (Lin et al., 2017; Chou et al., 2018). Results in the present study indicated that SAL significantly decreased the XOD activity in the liver of the HUA mice, but the inhibitory effect of SAL is much weaker than FBX. Hence, SAL was able to reduce UA production by decreasing the activity of XOD, which might be the effect of gallic acid. It was reported that gallic acid inhibits XOD *in vitro*. Moreover, urate transporters in the kidney are closely associated with UA excretion (Ichida et al., 2012). The excretion of UA includes urate reabsorption and secretion. URAT1 and GLUT9 mainly participate in urate reabsorption, whereas OAT1 is an important mediator for primary renal urate secretion (So and Thorens, 2010; Zhou et al., 2019). Results indicated a significant decrease in the protein levels of URAT1 and GLUT9 and a distinct increase in those of OAT1 in SAL-treated groups compared to the HUA group, which was consistent with previous studies. Moreover, the effects of 100 and 200 mg/kg SAL on the protein expression of GLUT9, URAT1, and OAT1 were superior to those of FBX, and the effect of 200 mg/kg SAL on URAT1 protein expression was stronger than that on the URAT1 inhibitor BZM. Summarily, SAL can not only reduce the production of uric acid by downregulating XOD activity in the liver but also promote the excretion of uric acid by regulating the protein expression of related renal uric acid transporters.

HUA has also been reported to be closely associated with oxidative stress (Tomiyama et al., 2018; Zhou et al., 2018). In the condition of HUA, excess UA is mainly distributed in vascular cells or adipocytes (Zhou et al., 2014). UA activates NADPH oxidase and subsequently produces ROS (Bergamini et al., 2009). Thus, to prevent HUA, it might be a feasible way to suppress the oxidative stress produced by UA. In the current study, it was confirmed that there was an oxidative imbalance in the kidney tissues of HUA mice. SAL significantly increased the activities of the antioxidant enzymes, including SOD, CAT, and GSH, and also suppressed the production of ROS and MDA. These results are in line with many studies (Hong et al., 2014; Wang et al., 2016; Mehmood et al., 2020). In addition, 200 mg/kg of SAL has a stronger effect on CAT, GSH activity, and MDA level than BZM, and its ameliorative effect on GSH activity is better than that of FBX. The results demonstrated that SAL significantly alleviated HUA by inhibiting renal oxidative stress.

**FIGURE 11:**
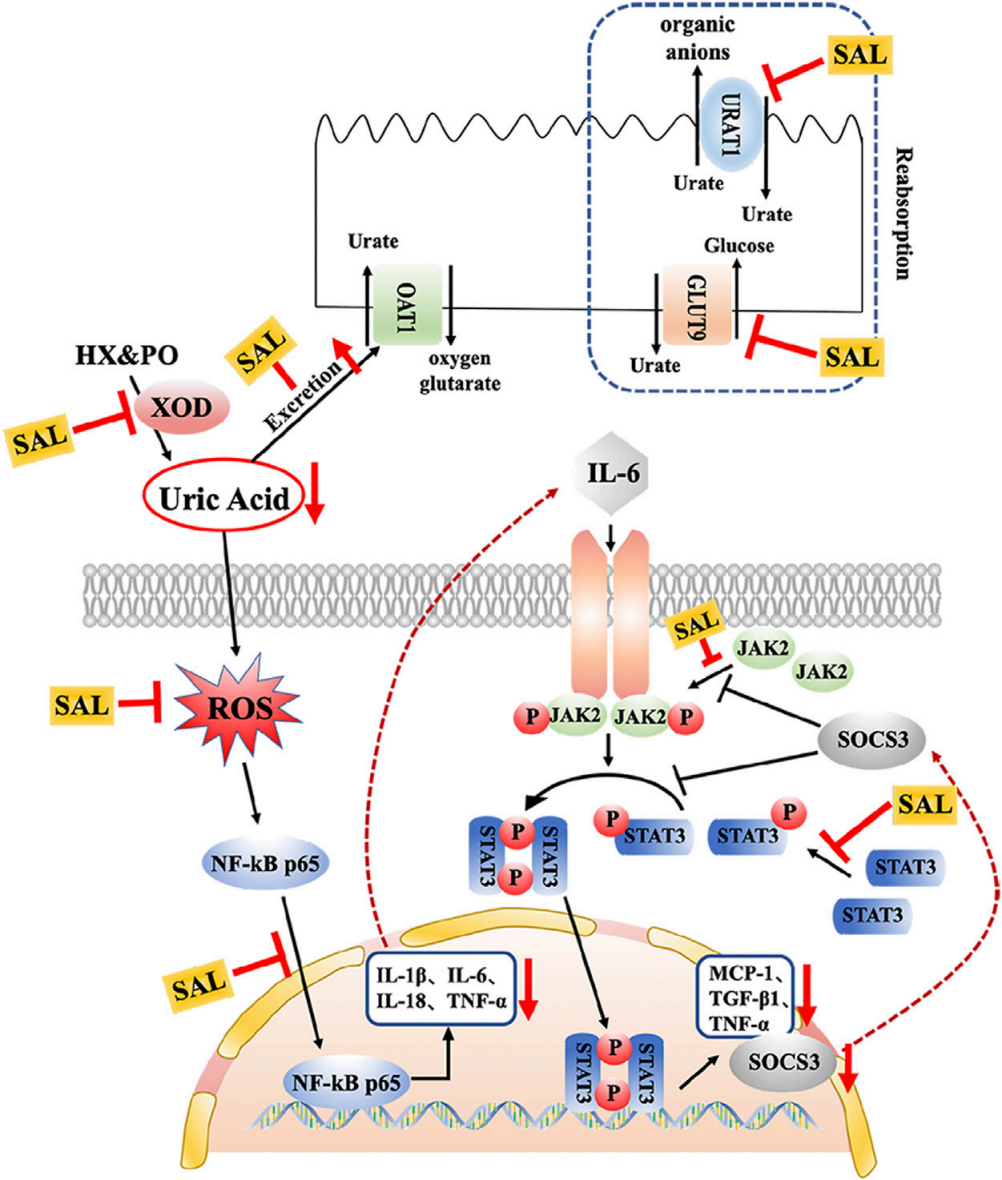
Schematic diagram of the proposed mechanisms of SAL protective effect against PO- and HX-induced HUA in mice.

In clinical practice, inseparably linked to oxidative stress, inflammation is a pathological feature of hyperuricemia and renal diseases (Joosten et al., 2020). Accumulation of UA triggers renal injury by production of pro-inflammatory cytokines. Besides, oxidative stress can also aggravate the inflammatory responses *via* activation of the NF-κB pathway (Mehmood et al., 2020). Thus, the effect against the activation of the NF-κB pathway might be related to the antioxidant activity. NF-κB p65 is reserved in an inactive form within the cytoplasm. When it is activated, it translocates into the nucleus and activates translation and transcription of pro-inflammatory cytokines, including TNF-α, IL-6, IL-18, and IL-1β (Baeuerle and Baltimore, 1996). In the present study, increases in the levels of TNF-α, IL-6, IL-18, and IL-1β were observed in HUA mice, which is consistent with previous studies (Isaka et al., 2016; Zhou et al., 2018). On the contrary, results of ELISA and qPCR analysis indicated that SAL supplement obviously diminished rises in expression and generation of TNF-α, IL-6, IL-18, and IL-1β in the HUA mice. Moreover, interleukin receptors can activate the JAK2 receptor, which activates the kinase function of JAK2, leading to phosphorylation (Takizawa et al., 2001). Subsequently, STAT3 protein binds to the phosphorylated receptor, where STAT3 is phosphorylated by JAK2. Finally, the p-STAT3 protein translocates into the nucleus and is involved in the signal transduction and gene regulation of a variety of important inflammatory cytokines, including SOCS3, TGF-β, and MCP-1. MCP-1, an inflammatory factor secreted by monocytes or macrophages, participates in the pathological process of glomerulonephritis (Chen et al., 2019). Additionally, SOCS3 negatively regulated the JAK/STAT signaling pathway (Simon et al., 2020; Xin et al., 2020). Previous studies have shown that the JAK/STAT signaling pathway in HUA mice was activated and the level of SOCS3 was overexpressed. After the induction of PO and HX, the phosphorylation level of JAK2 and STAT3 in the kidney of HUA mice was significantly increased, and the mRNA level of SOCS3 was significantly increased. However, the treatment with SAL significantly suppressed JAK2 activation and STAT3 phosphorylation. Besides, SAL treatment decreased the gene expression of SOCS3, TGF-β, and MCP-1, showing that SAL can inhibit the activation of the JAK2-STAT3 signaling pathway. Based on previous literature (Rosa et al., 2016; Adachi et al., 2019), it was deduced that anti-inflammatory active ingredient of SAL might be vitexin and isorhamnetin. In conclusion, this study demonstrated that SAL ameliorated HUA *via* suppressing the JAK/STAT signaling pathway (Figure 11).

However, does SAL suppress renal oxidative stress and inflammatory response by inhibiting UA production or promoting excretion? In our study, we found that SAL not only inhibited UA production but also facilitated UA excretion. SAL could inhibit XOD activity, but not as much as FBX. SAL played a crucial role in promoting UA excretion in mice with HUA, whose effect was much stronger than BZM. It thus seems plausible that improvement of renal damage in HUA by SAL might be due to the regulatory role in UA metabolism.

## Conclusion

In conclusion, our study demonstrated the anti-hyperuricemic and nephroprotective effects of SAL in PO/HX-induced HUA mice. The anti-hyperuricemia effect of SAL attributed to the dual roles of regulating the UA production and excretion. Furthermore, SAL possessed nephroprotective effects *via* attenuation of the HUA-induced oxidative stress and inflammatory reaction, which was related to its ability to inhibit the JAK/STAT/NF-κB signaling pathway. This evidence suggests that SAL should be considered in the development of novel chemopreventive or chemotherapeutic agent for HUA and UA nephropathy and tested in further clinical studies of novel drug development.

## Data Availability

The original contributions presented in the study are included in the article/Supplementary Material. Further inquiries can be directed to the corresponding authors.
