# Corrigendum: The Extract of *Sonneratia apetala* Leaves and Branches Ameliorates Hyperuricemia in Mice by Regulating Renal Uric Acid Transporters and Suppressing the Activation of the JAK/STAT Signaling Pathway

**DOI:** 10.3389/fphar.2021.760098

**Published:** 2021-10-15

**Authors:** Yu-Lin Wu, Jin-Fen Chen, Lin-Yun Jiang, Xiao-Li Wu, Yu-Hong Liu, Chang-Jun Gao, Yan Wu, Xiao-Qing Yi, Zi-Ren Su, Jian Cai, Jian-Nan Chen

**Affiliations:** ^1^ Guangdong Provincial Key Laboratory of New Drug Development and Research of Chinese Medicine, School of Pharmaceutical Sciences, Guangzhou University of Chinese Medicine, Guangzhou, China; ^2^ The First Affiliated Hospital of Chinese Medicine, Guangzhou University of Chinese Medicine, Guangzhou, China; ^3^ School of Biomedical and Pharmaceutical Sciences, Guangdong University of Technology, Guangzhou, China; ^4^ Guangdong Academy of Forestry, Guangzhou, China; ^5^ Guangdong Provincial Key Laboratory of Silviculture, Protection and Utilization, Guangzhou, China

**Keywords:** *Sonneratia apetala* leaves and branches, hyperuricemia, renal uric acid transporters, oxidative stress, JAK/STAT pathway

In the original article, there was a mistake in Table 2 as published. During the process of review, we modified the content (“Identification”) of the table. But during proofing stage, incorrect version of table was provided because of our carelessness. The corrected Table 2 appears below.

**TABLE 2:** Identification of the chemical constituents in SAL.

Number	Retention time (min)	Ion mode	Extraction mass (Da)	Found mass (Da)	Error (ppm)	Formula	Identification	Peak area (%)
1	1.61	−	134.0200	134.0201	0.6715	C_4_H_6_O_5_	L-(-)-Malic acid	0.7299
2	4.18	+	153.1159	153.1152	−4.9636	C_9_H_17_NO_2_	(4E)-3-Hydroxy-2,4-dimethyl-4-heptenamide	8.8478
3	4.82	−	170.0205	170.0203	−1.5880	C_7_H_6_O_5_	Gallic acid	9.8934
4	10.79	+	178.0994	178.0992	−0.7861	C_11_H_14_O_2_	4-Isobutylbenzoic acid	0.2395
5	10.86	−	335.1158	335.1153	−1.3428	C_20_H_17_NO_4_	Berberine	0.1830
6	12.75	+	150.1045	150.1044	−0.5330	C_10_H_14_O	Carvone	0.1451
7	13.54	−	316.0583	316.0582	−0.2848	C_16_H_12_O_7_	Isorhamnetin	6.5621
8	13.89	+	432.1057	432.1052	−1.1571	C_21_H_20_O_10_	Vitexin	1.8300
9	16.25	−	132.0575	132.0577	1.5145	C_9_H_8_O	trans-Cinnamaldehyde	0.1165
10	16.29	+	314.2457	314.2454	−1.1456	C_18_H_34_O_4_	(+/−)12(13)-DiHOME	2.8721
11	18.99	+	350.2063	350.2064	0.2570	C_20_H_30_O_5_	Andrographolide	0.2457
12	21.40	−	337.3345	337.3337	−2.2233	C_22_H_43_NO	Erucamide	0.3520

The authors apologize for this error and state that this does not change the scientific conclusions of the article in any way. The original article has been updated.

